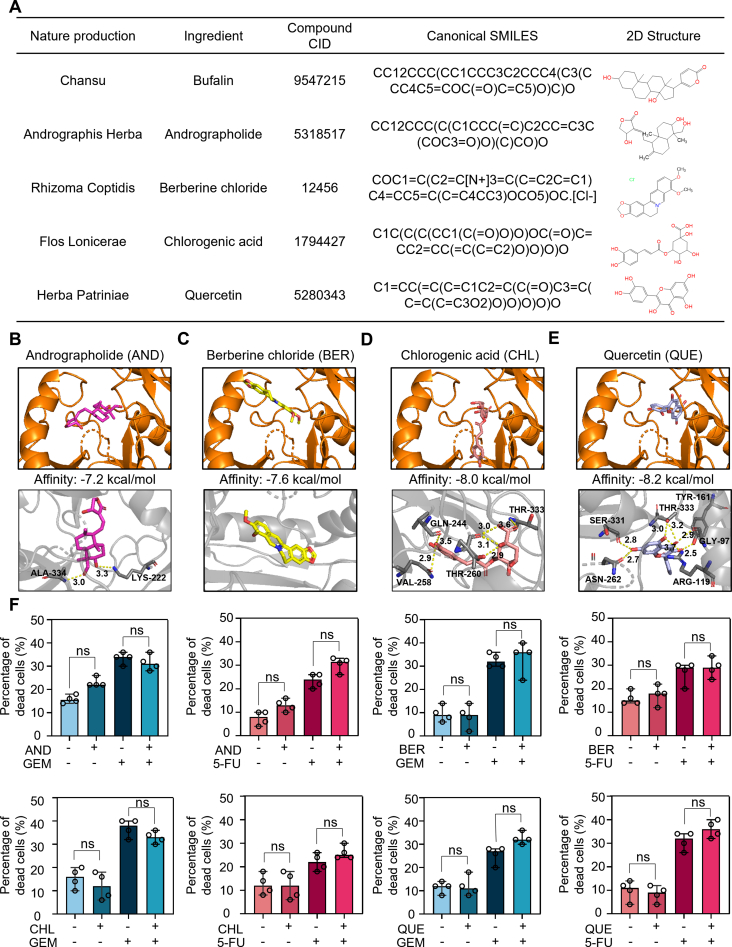# A novel BCAT1 inhibitor bufalin sensitizes pancreatic cancer cells to chemotherapy

**DOI:** 10.1016/j.gendis.2024.101503

**Published:** 2024-12-24

**Authors:** Wei Zhang, Yibao Fan, Milad Ashrafizadeh, Dan Shi, Gautam Sethi, Yavuz Nuri Ertas, A.M. Abd El-Aty, Xianbin Zhang, Si Chen, Peng Gong

**Affiliations:** a Department of General Surgery, Institute of Precision Diagnosis and Treatment of Digestive System Tumors and Guangdong Provincial Key Laboratory of Chinese Medicine Ingredients and Gut Microbiomics, Carson International Cancer Center, Shenzhen University General Hospital, Shenzhen University, Shenzhen, Guangdong 518055, China; b School of Pharmacy, Shenzhen University Medical School, Shenzhen, Guangdong 518060, China; c Department of Pharmacology and NUS Centre for Cancer Research, Yong Loo Lin School of Medicine, National University of Singapore, Singapore 117600, Singapore; d Department of Biomedical Engineering, Erciyes University, Kayseri 38039, Turkey; e Department of Technical Sciences, Western Caspian University, AZ1001, Baku, Azerbaijan; f Department of Pharmacology, Faculty of Veterinary Medicine, Cairo University, Giza 12211, Egypt; g Department of Medical Pharmacology, Medical Faculty, Ataturk University, Erzurum 25240, Turkey; h Department of Immunology, Shenzhen University Medical School, Shenzhen, Guangdong 518060, China

Pancreatic cancer is one of the most lethal cancers worldwide and is characterized by a poor prognosis.^1^^,^^2^ Due to its aggressive nature and lack of early symptoms, most patients are diagnosed at an advanced stage, and chemotherapy is the optimal option.^3^^,^^4^ Epidemiology, the incidence rate of pancreatic cancer has increased in the last two decades, from 196,000 patients in 1990 to 441,000 in 2017. Based on 2020 global cancer statistics, the annual cases of pancreatic cancer have increased to 195,773.^4^ Unfortunately, patients demonstrate resistance to chemotherapy, and only some of them benefit from current therapeutic strategies.^5^ Systemic chemotherapy has been widely applied for the treatment of pancreatic cancer. However, inter- and intratumoral heterogeneity, especially changes in genomic and immunological profiles, can lead to therapeutic resistance. Moreover, *SMAD4*, *BRCA1/2*, and *PALB2* mutations have been associated with therapy resistance in pancreatic cancer.^4^ This highlights the urgent need to explore the underlying mechanisms of chemoresistance and to develop innovative therapeutic strategies.

To investigate the genes involved in chemoresistance, we obtained 179 pancreatic cancer tissues and 171 normal tissues from The Cancer Genome Atlas (TCGA) and The Genotype-Tissue Expression Project (GTEx). We observed that 7903 genes were up-regulated and 369 genes were down-regulated (Fig. S1A, B). Subsequently, we determined the genes associated with overall survival, disease-free interval, disease-specific survival, and progression-free interval, respectively. We noted that 429 genes (416 up-regulated genes and 13 down-regulated genes) were associated with the survival of patients (Fig. S1C, D). Next, we evaluated the prognostic values of these genes with the support of a 5-fold cross-validated least absolute shrinkage and selection operator (LASSO) regression model and it was repeated 1000 times on different seeds. The frequency of genes that significantly controlled the survival of patients is presented in Fig. S1E. To eliminate the confounding effects of these genes, univariate and multivariate Cox regression analyses were performed (Fig. S1F). We observed that Wnt family member 7A (WNT7A), branched-chain amino acid transaminase 1 (BCAT1), lymphocyte antigen 6 family member D (LY6D), and UBX domain protein 2A (UBXN2A) were the independent risk factors for survival (Fig. S1F). High expression of WNT7A (Fig. S2A–D), LY6D (Fig. S2E–H), UBXN2A (Fig. S2I–L), and BCAT1 (Fig. 1A–D) significantly decreased overall survival, disease-free interval, disease-specific survival, and progression-free interval.

**Figure 1:**
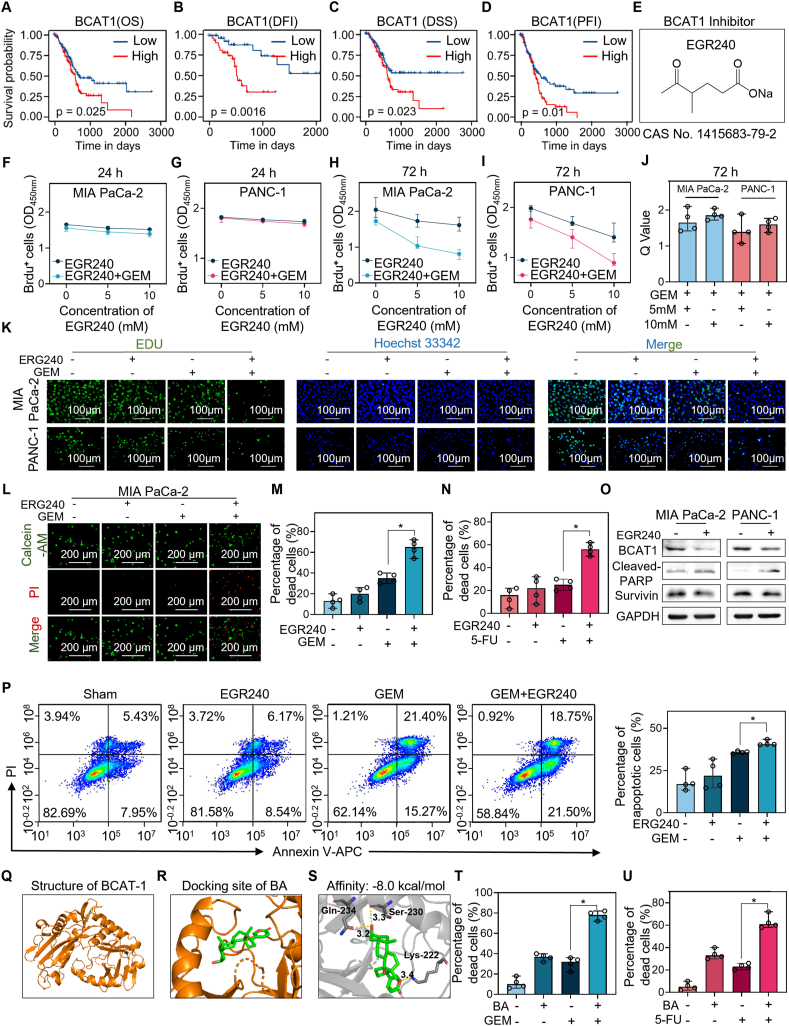
Bufalin inhibits BCAT1 and sensitizes pancreatic cancer cells to GEM and 5-FU. **(A)** Kaplan–Meier overall survival (OS) analysis, **(B)** disease-free interval (DFI) analysis, **(C)** disease-specific survival (DSS) analysis, and **(D)** progression-free interval (PFI) analysis for PDAC patients with high versus those low expression of branched-chain amino acid transaminase 1 (BCAT1) suggest that high expression of BCAT1 is associated with poor prognosis. **(E)** EGR240 is the sole inhibitor of BCAT1. **(F, G)** MIA PaCa-2(F) and PANC-1(G) cells with vehicles, EGR240, gemcitabine (GEM) and indicated drug combinations for 24 h. However, we did not observe proliferative activity. **(H, I)** Only after incubating for 72 h, we can observe that the combination of 5 mM or 10 mM EGR240 with GEM significantly enhanced the antiproliferative effect of GEM in both MIA PaCa-2 cells (H) and PANC-1 cells (I). **(J)** Q value was calculated to assess the synergistic effects of EGR240 and GEM. **(K, L)** EdU staining (K) and calcein-AM/PI (L) double staining assays verified the findings under a fluorescence microscope. **(M, N)** Trypan blue results indicated that inhibition of BCAT1 significantly induced cell death in combination with GEM (M) and 5-FU (N). **(O)** Western blotting showed changes in the level of apoptotic protein cleaved PARP and antiapoptotic protein Survivin in MIA PaCa-2 and PANC-1 cells. **(P)** The number of apoptotic cells when treated with vehicles, EGR240, GEM or the drug combination were determined with the assistance of Annexin V-APC/PI apoptotic staining. **(Q, S)** Molecular docking analysis between bufalin and BCAT1 (Q), and the binding affinity was determined (S). **(T, U)** Percentage of dead cells significantly increased in drug combination group compared to the monotherapy group.

To investigate if and how BCAT1 was involved in chemoresistance, MIA PaCa-2 cells or PANC-1 cells were treated with EGR240, the sole inhibitor of BCAT1 (Fig. 1E–G), for 24 h and 72 h (Fig. 1H, I). We observed a synergistic effect of EGR240 and gemcitabine on inhibiting cell proliferation after 72 h of treatment (Fig. 1J). EdU assays also suggested that inhibition of BCAT1 enhanced the antiproliferative activity of gemcitabine (Fig. 1K). Additionally, we evaluated the anticarcinoma activity of the combination of EGR240 and gemcitabine by calcein-AM/propidium iodide (PI) staining, and we observed that compared with monotherapy, the combination of EGR240 and gemcitabine significantly increased the percentage of PI^+^ cells, which represent dead cells (Fig. 1L). This finding was verified by the trypan blue assay, which indicated that EGR240 significantly enhanced the cell death induced by gemcitabine (Fig. 1M) and 5-fluorouracil (Fig. 1N). To investigate apoptosis, Western blot assays were performed. We observed that inhibition of BCAT1 increased the level of the apoptotic protein cleaved poly (ADP-ribose) polymerase (cleaved-PARP) and decreased the level of the antiapoptotic protein survivin (Fig. 1O). Indeed, the annexin V-APC/PI assay demonstrated that the combinational therapy significantly increased the number of apoptotic cells compared to vehicle or monotherapy (Fig. 1P). Additionally, EGR240 treatment significantly elevated the expression levels of p62 and LC3II in both MIA PaCa-2 (Fig. S3A, B) and PANC-1 (Fig. S3C, D) cells, compared to those in sham group, thereby suggesting blockage of autophagy. These data suggest that inhibition of BCAT1 significantly increases the sensitivity of pancreatic cancer cells to chemotherapies.

Currently, to our knowledge, EGR240 is the sole inhibitor of BCAT1; however, it slightly inhibited cell proliferation only at high concentrations (5 mM and 10 mM) after prolonged incubation for 72 h (Fig. 1H, I). We, therefore, sought to identify potent and specific small-molecule inhibitors of BCAT1. First, we evaluated the relationships between BCAT1 and several natural ingredients being investigated in our laboratory, such as andrographolide, berberine chloride, bufalin, chlorogenic acid, and quercetin. These traditional Chinese medicines showed excellent antitumor effects in patients presenting a damp-heat pattern described in the traditional Chinese medicine system and significantly increased survival of patients. The docking pocket of the BCAT1 protein was constructed based on the binding sites of the cocrystal ligands (Fig. 1Q). We observed that all these drugs could bind to BCAT1 (Fig. S4A). The affinities were −7.2 kcal/mol for andrographolide (Fig. S4B), −7.6 kcal/mol for berberine chloride (Fig. S4C), −8.0 kcal/mol for chlorogenic acid (Fig. S4D), −8.2 kcal/mol for quercetin (Fig. S4E), and −8.0 kcal/mol for bufalin (Fig. 1R, S). The relevant amino acid residues are listed in Table S1. Subsequently, the benefits of these natural ingredients in treating pancreatic cancer were evaluated (Fig. S4F). We observed that among the treatments with the same dose of andrographolide, berberine chloride, chlorogenic acid, quercetin, or bufalin, only bufalin significantly increased the sensitivity of pancreatic cancer cells to gemcitabine (Fig. 1T) and 5-fluorouracil (Fig. 1U). Further assessment of cell viability in MIA PaCa-2 and PANC-1 cells treated with bufalin, the identified inhibitor of BCAT1, revealed IC_50_ values of 0.26 ± 0.046 μM and 0.32 ± 0.086 μM, respectively, indicating an effective outcome in pancreatic cancer (Fig. S5).

In conclusion, the present study demonstrated that BCAT1 contributes to chemoresistance in pancreatic cancer cells. Moreover, with the support of molecular docking, we demonstrate that bufalin directly binds to BCAT1 and increases the sensitivity of pancreatic cancer cells to gemcitabine and 5-fluorouracil. These results suggest that treatment with bufalin is an effective strategy for overcoming chemoresistance.

## Ethics declaration

The gene sequencing data of human tissues were obtained from the public databases The Cancer Genome Atlas (TCGA) and The Genotype-Tissue Expression Project (GTEx). An ethical approval statement is not necessary for this case.

## Author contributions

**Wei Zhang:** Validation, Writing- original draft, Writing- review & editing. **Yibao Fan:** Formal analysis, Validation. **Milad Ashrafizadeh:** Formal analysis, Validation, Writing - original draft. **Dan Shi:** Data curation. Formal analysis. **Gautam Sethi:** Formal analysis, Validation, Writing - review & editing. **Yavuz Nuri Ertas:** Formal analysis, Validation, Writing-review & editing. **A.M. Abd El-Aty:** Formal analysis, Validation, Writing - review & editing. **Xianbin Zhang:** Conceptualization, Supervision, Writing- original draft. **Si Chen:** Conceptualization, Supervision, Investigation. **Peng Gong:** Conceptualization, Investigation, Supervision, Writing - review & editing.

## Conflict of interests

The authors declared no conflict of interests.

## Funding

This study was supported by the 10.13039/501100001809National Natural Science Foundation of China (No. 82405167), the Shenzhen Science and Technology Program (Guangdong, China) (No. GJHZ20220913143005010), and the fundamental research project of the Shenzhen Science and Technology Innovation Commission (No. 20231120113324002 and No. RCBS20231211090635056).

## Figures and Tables

**figs1:**
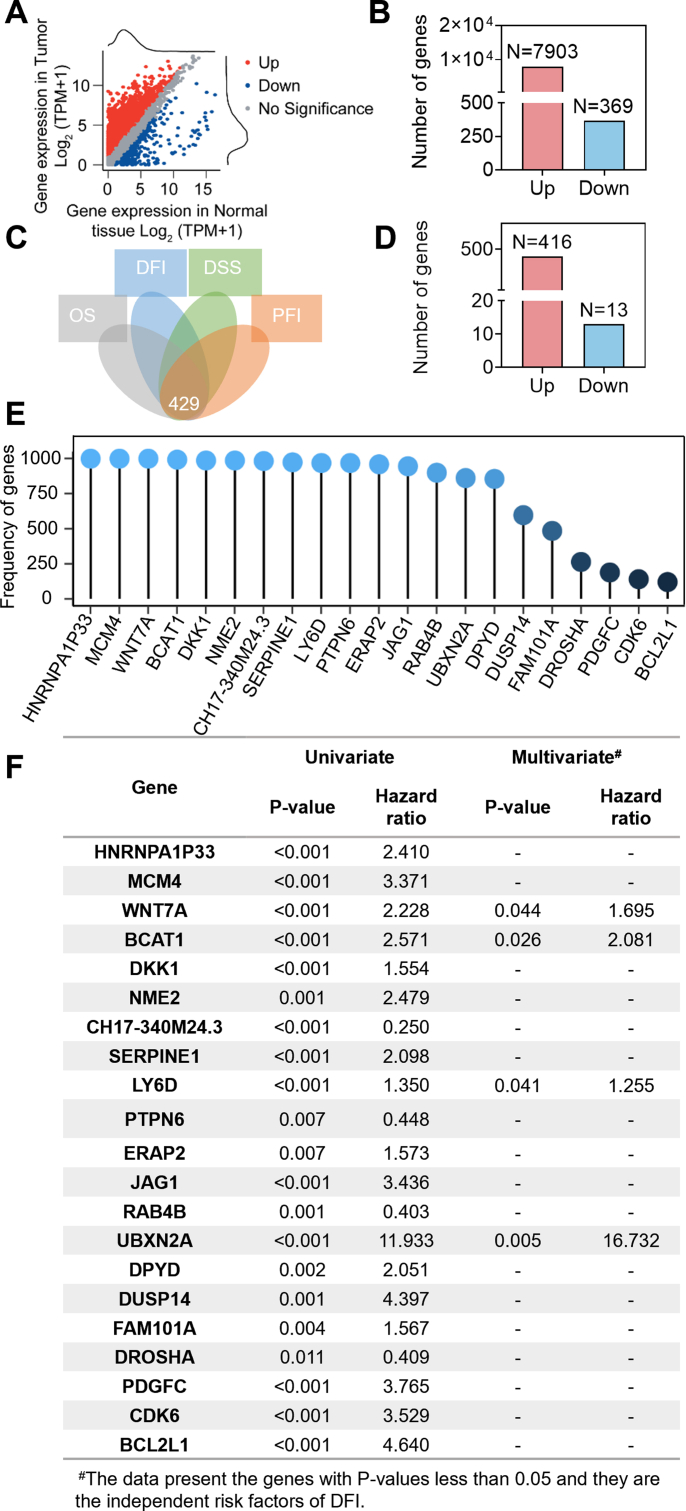


**figs2:**
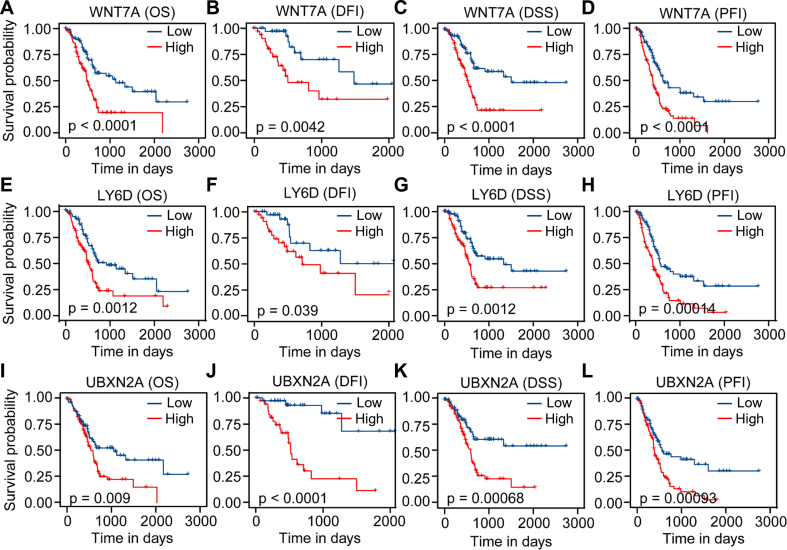


**figs3:**