# Supplementary material for: A novel BCAT1 inhibitor bufalin sensitizes pancreatic cancer cells to chemotherapy
Source: Genes Dis. 2024 Dec 24;12(3):101503. doi: 10.1016/j.gendis.2024.101503 (PMC11803225; doi:10.1016/j.gendis.2024.101503)
Supplement: Multimedia component 1 [file mmc1.docx]

**Materials and methods**

**Clinical cohort**

To determine the genes contributing to prognosis and chemoresistance, we obtained clinical information of pancreatic cancer patients from The Cancer Genome Atlas (TCGA) via the UCSC Xena project (http://xena.ucsc.edu/). The differential expression of genes in pancreatic tissues and pancreatic cancer tissues was evaluated with the support of the “limma” package, and the survival of patients was determined by Kaplan‒Meier curves with the support of the “survival” and “survminer” packages.

**Cell culture and reagents**

The human pancreatic cancer cell lines PANC-1 and MIA PaCa-2 were purchased from the China Center for Type Culture Collection (CCTCC). These cells were cultured in Dulbecco’s modified Eagle’s medium (DMEM, HyClone, Logan, USA, catalog no. SH30243.01) supplemented with 10% fetal bovine serum (FBS, AusGeneX, Gold Coast, Australia, catalog no. FBS500-S), 100 units/mL penicillin and 100 μg/mL streptomycin (Gibco, New York, USA, catalog no. 15140-122). Gemcitabine (GEM, catalog no. S1149), 5-fluorouracil (5-FU, catalog no. S1209), bufalin (BA, catalog no. S7821), andrographolide (AND, catalog no. S2261), berberine chloride (BER, catalog no. S9046), chlorogenic acid (CHL, catalog no. S2280) and quercetin (QUE, catalog no. S2391) were obtained from Selleck Chemicals (Houston, USA). ERG240 was purchased from MedChemExpress (Monmouth Junction, USA, catalog no. HY-W193545A).

**Evaluation of cell proliferation**

For assessment of cell proliferation, 2×10^3^ MIA PaCa-2 or PANC-1 cells per well were seeded in a 96-well plate. The cells were incubated with vehicles, 5 mM EGR240, 10 mM EGR240, 10 μM GEM, or the drug combination for 24 h or 72 h. Cell proliferation was evaluated by quantifying the incorporation of 5-bromo-2´-deoxyuridine (BrdU) with a colorimetric cell proliferation ELISA kit (Sigma‒Aldrich, catalog no. 11647229001). The absorbance was measured at 450 nm to determine the number of BrdU^+^ cells (proliferative cells) using a Synergy LX micromode reader (BioTek, Winooski, USA). The Q value was calculated as follows: Q value= (inhibition rate of EGR240 plus GEM)/ (inhibition rate of EGR240 + inhibition rate of GEM- inhibition rate of EGR240 × inhibition rate of GEM). To observe the proliferative cells intuitively, EDU^+^ cells stained with the BeyoClick™ EdU Cell Proliferation Kit with Alexa Fluor 488 (Beyotime, Shanghai, China, code: C0071) were stained green and observed with the support of an Axio Vert. A1 fluorescence microscope (Carl Zeiss, Oberkochen, Germany). To assess cell viability, 4×10^3^ MIA PaCa-2 or PANC-1 cells per well were seeded in a 96-well plate. Cells were incubated with BA at the indicated concentrations for 24 h. Cell viability was evaluated by the cell counting kit-8 (CCK-8, MedChemExpress, catalog no. HY-K0301), and the absorbance was determined at 450 nm using a Synergy LX multimode reader.

**Assessment of cell death**

To evaluate cell death, 2.5×10^5^ MIA PaCa-2 cells per well were seeded in a 6-well plate. The cells were treated with vehicles, 5 mM EGR240, 10 μM GEM or 5 mM EGR240 plus 10 μM GEM for 72 h. Subsequently, the cells were stained according to the instructions of the calcein AM/propidium iodide (PI) double staining assay (Yeasen, catalog no. 40747ES76), and fluorescence images were captured using an Axio Vert. A1 fluorescence microscope. In addition, 3×10^4^ MIA PaCa-2 or PANC-1 cells were plated in a 24-well plate. On the next day, the cells were incubated with vehicles, 5 mM EGR240, 0.5 μM BA, 0.5 μM AND, 0.5 μM CHL, 0.5 μM BER, 0.5 μM QUE, 10 μM GEM, 100 μM 5-FU or the drug combination for 24 h. The dead cells were stained with trypan blue (Solarbio, Beijing, China, code C0040), and the percentage of dead cells was calculated as follows: percentage of dead cells (%) = 100%× trypan blue^+^ cells/ (trypan blue^+^ cells + trypan blue^-^ cells).

To evaluate how EGR240 induces cell death, 2.5×10^5^ MIA PaCa-2 and PANC-1 cells per well were seeded per well, and the cells were treated with 5 mM EGR240 and 5 mM EGR240 plus 10 μM GEM. Western blotting was performed using the following antibodies: anti-cleaved PARP (Cell Signaling, Danvers, USA, catalog no. 5625, dilution: 1000×), anti-survivin (Cell Signaling, catalog no. 2808, dilution: 1000×), anti-GAPDH (ABclonal, catalog no. AC001, dilution: 1000×), anti-SQSTM1/p62 (ABclonal, catalog no. A19700, dilution: 1000×), anti-LC3B (ABclonal, catalog no. A19665, dilution: 1000×) and β-Actin-peroxidase antibody (Sigma-Aldrich, catalog no. A3854, dilution: 40000×). To determine the number of apoptotic cells, the cells were also stained with an Annexin V-APC/PI apoptosis kit (Elabscience, Houston, USA, catalog no. E-CK-A217), and the percentage of apoptotic cells stained was determined with the help of a NovoCyte flow cytometer system (Agilent, Santa Clara, USA) using the following formula: percentage of apoptotic cells (%) = 100% × (Annexin V-APC^+^PI^-^+Annexin V-APC^+^PI^+^) cells/total cells.

**Molecular docking**

The crystal structure of the BCAT1 protein was retrieved from the Protein Data Bank (<https://www.rcsb.org/structure/7NWA>), and 3D structures of andrographolide (compound CID: 5318517), berberine chloride (compound CID: 12456), bufalin (compound CID: 9547215), chlorogenic acid (compound CID: 1794427) and quercetin (compound CID: 5280343) were obtained from PubChem. Molecular docking was performed using AutoDock Vina based on docking pockets constructed by the AutoDock tool centered at -38.91 Å, 3.628 Å, and 17.325 Å with dimensions of 22.5 Å×22.5 Å× 22.5 Å. Subsequent molecular interaction analysis and visualization were subsequently performed by PyMOL.

**Statistical analysis**

The relationship between gene expression and survival was determined by Kaplan‒Meier survival curves. Subsequently, the independent prognostic factors were evaluated by univariate and multivariate Cox proportional hazard models. For the in vitro experiments, the data are presented as medians with 95% confidence intervals (CIs), and the Mann‒Whitney rank-sum test followed by Bonferroni correction was used to determine significant differences. A *p* value lower than 0.05 was considered significant.

**
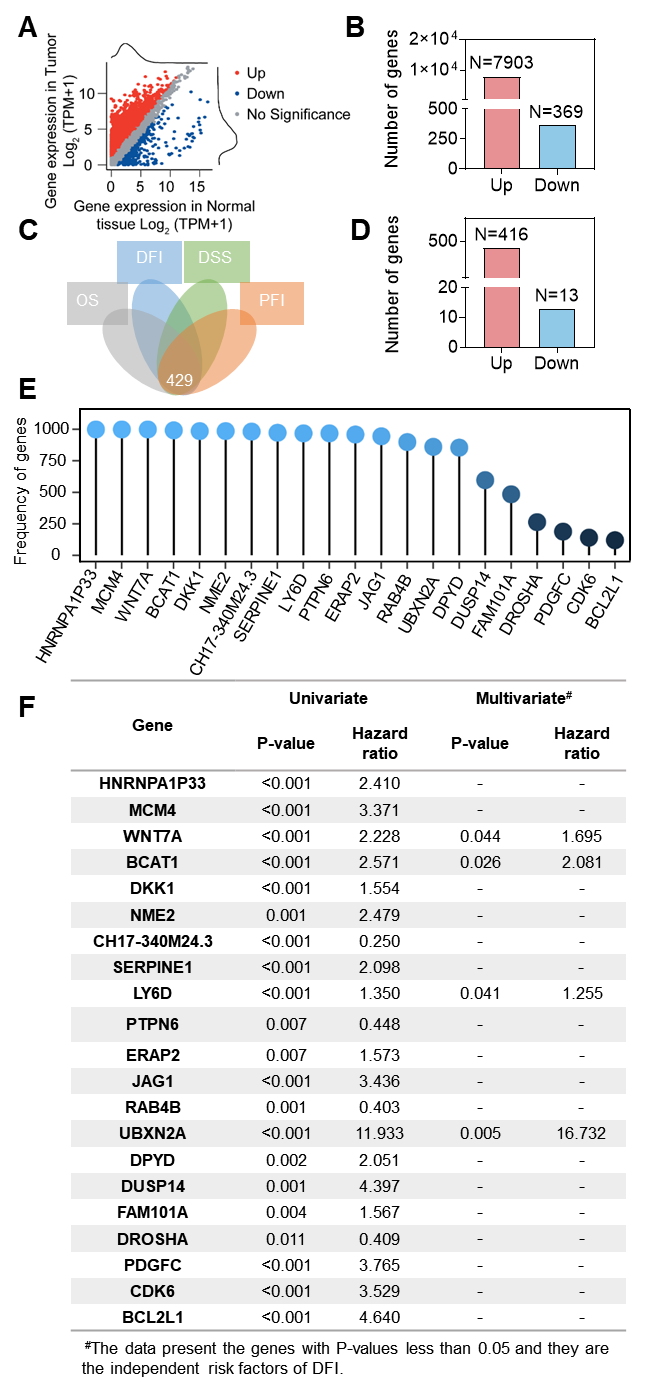
**

**Figure S1. Identifying genes associated with** **survival of patient.** (A) The volcano plot shows the landscape of gene expression. (B) Histogram showing differentially expressed genes (DEGs); (C) The genes are associated with overall survival (OS), disease-free interval (DFI), disease-specific survival (DSS), and progression-free interval (PFI). (D) Histogram presents genes associated with survival; (E) The frequency of genes that appeared at least 50 times during 1000 fits. (F) The genes associated with the DFI were determined by univariate and multivariate Cox regression analyses.


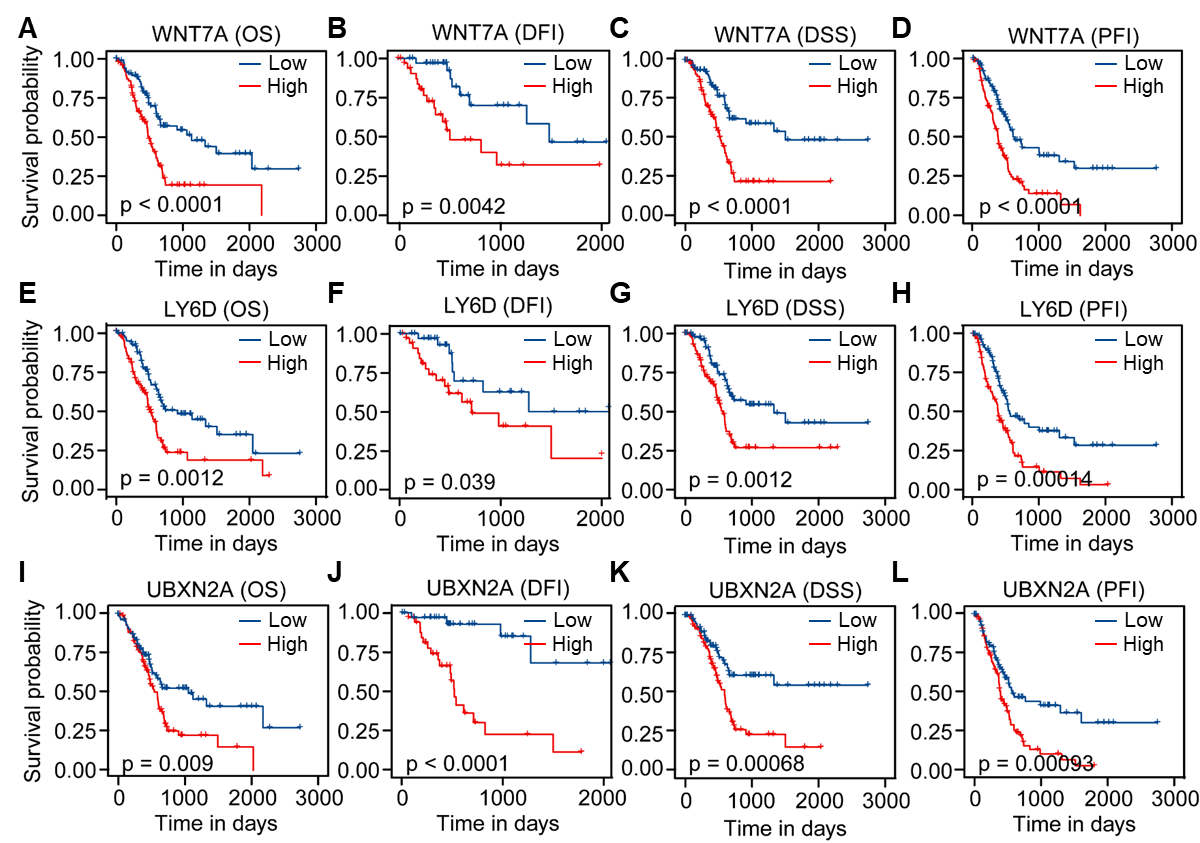


**Figure S2. Validation of the relationships between genes and the OS, DFI, DSS or PFI.** High expression of (A-D) Wnt family member 7A (WNT7A), (E-H) lymphocyte antigen 6 family member D (LY6D) and (I-L) UBX domain protein 2A (UBXN2A) significantly decreased the overall survival (OS), disease-free interval (DFI), disease-specific survival (DSS) and progression-free interval (PFI) in pancreatic cancer patients.


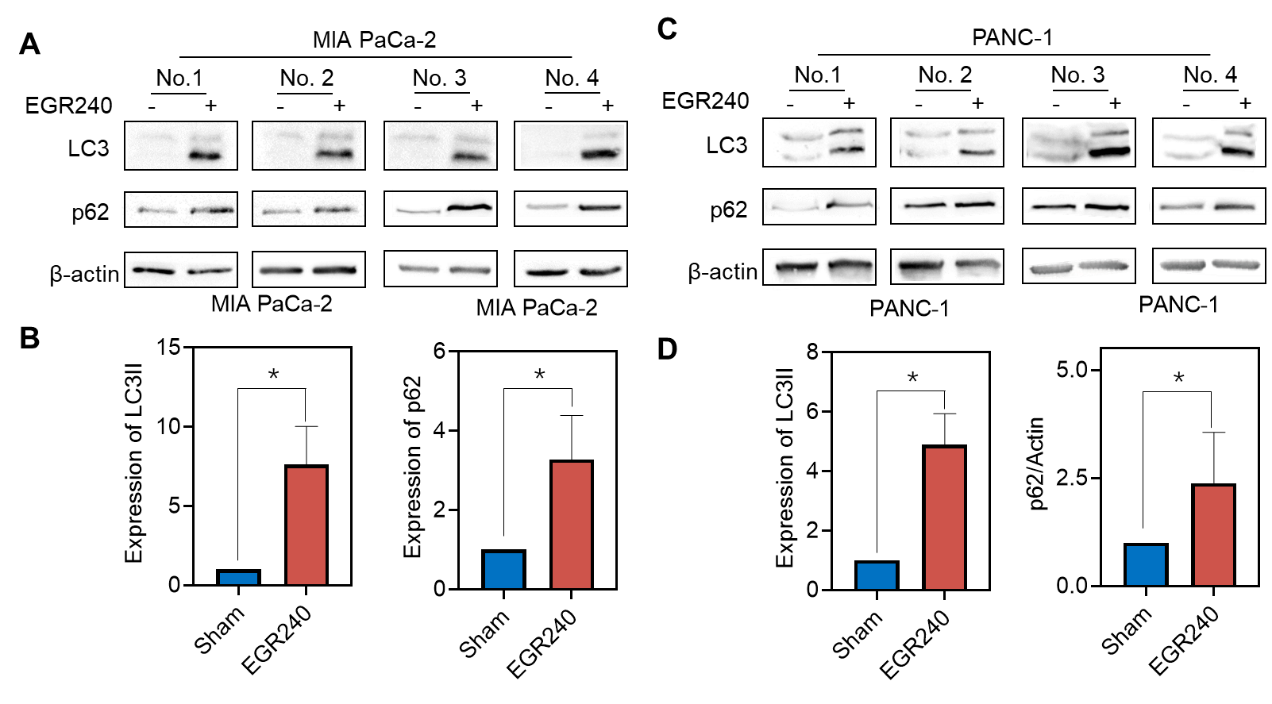


**Figure S3. Inhibition of BCAT1 by EGR240 treatment blocks autophagy.** Western blotting of LC3 and p62 in (A, B) MIA PaCa-2 and (C, D) PANC-1 cells.


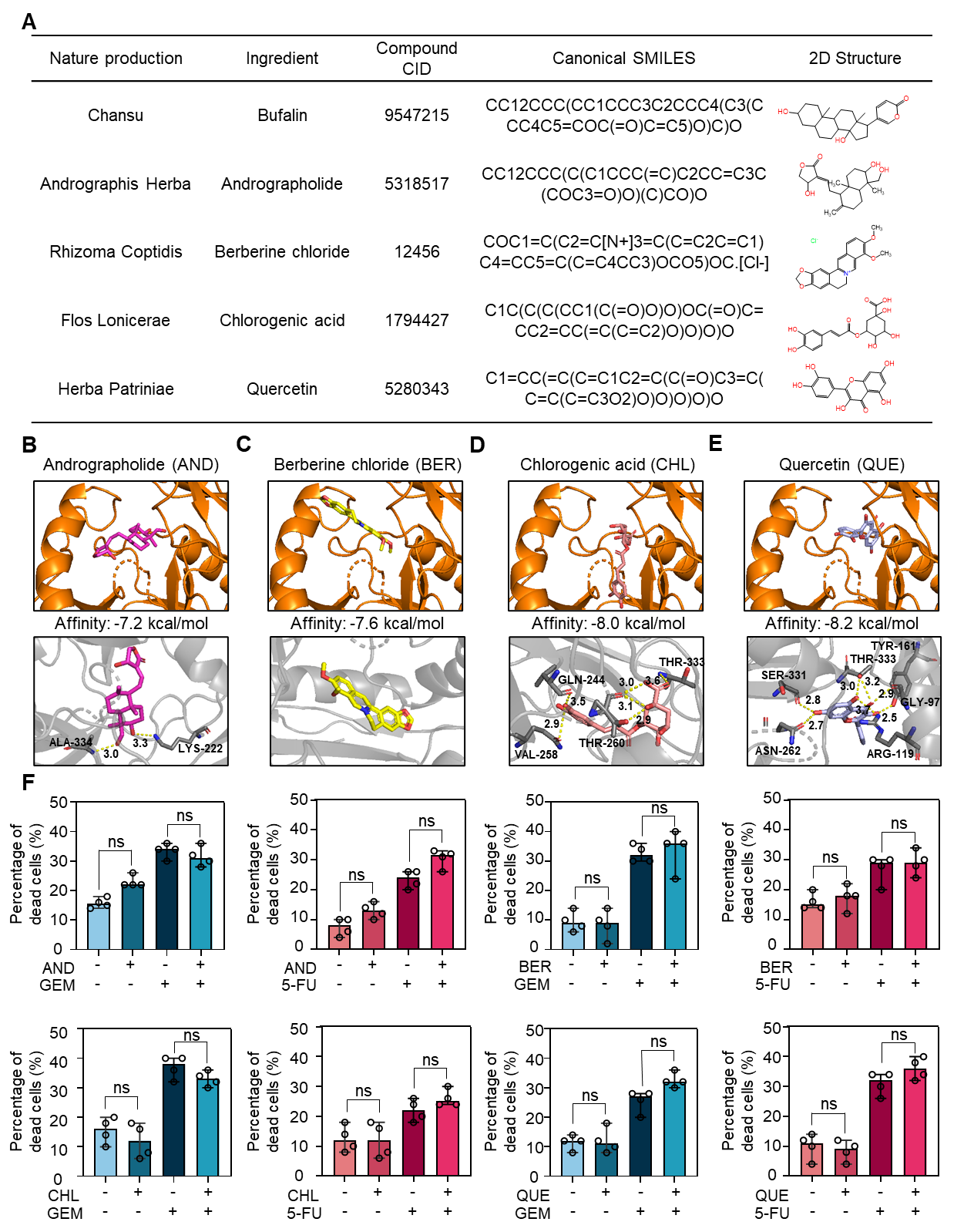


**Figure S4. Screening and identification of BCAT1 inhibitors.** (A) Several natural product-derived ingredients were examined for BCAT1 inhibition. Molecular docking was performed to evaluate the interactions between BCAT1 and (B) andrographolide (AND), (C) berberine chloride (BER), (D) chlorogenic acid (CHL) or (E) quercetin (QUE). (F) Trypan blue assays indicated that neither AND, BER, CHL nor QUE significantly improved the antitumor effect of gemcitabine (GEM) or 5-fluorouracil (5-FU). ns represents no significance.


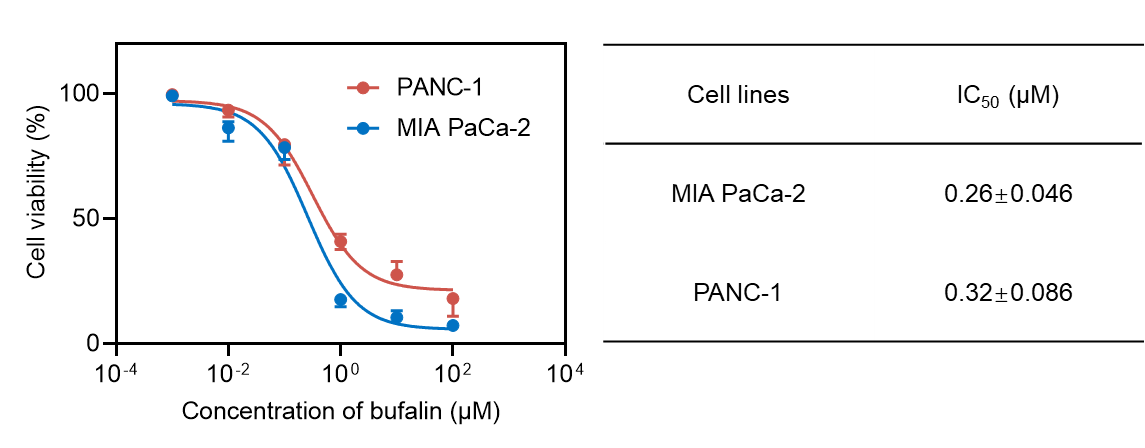


**Figure S5. Assessment of the antitumor effect of bufalin, the newly identified inhibitor.** CCK-8 assay was performed after treating PANC-1 or MIA PaCa-2 with bufalin at the indicated concentrations.

| Small molecules | | Amino acid residues of BCAT-1 | (Å) |
| --- | --- | --- | --- |
| Bufalin | Lys-222 | | 3.4 |
| Bufalin | Gln-234 | | 3.2 |
| Bufalin | Ser-230 | | 3.3 |
| Andrographolide | Ala-334 | | 3.0 |
| Andrographolide | Lys-222 | | 3.3 |
| Quercetin | Tyr-161 | | 2.5 |
| Quercetin | Gly-97 | | 2.9 |
| Quercetin | Arg-119 | | 3.7 |
| Quercetin  Quercetin | Thr-333  Thr-333 | | 3.0  3.2 |
| Quercetin | Ser-331 | | 2.8 |
| Quercetin | Asn-262 | | 2.7 |
| Chlorogenic acid | Val-258 | | 2.9 |
| Chlorogenic acid  Chlorogenic acid  Chlorogenic acid  Chlorogenic acid | Gln-244  Thr-260  Thr-260  Thr-260 | | 3.5  3.1  2.9  3.0 |
| Chlorogenic acid | Thr-333 | | 3.6 |

Table S1. The distance between the amino acid residues of BCAT1 and traditional Chinese ingredients.
